# Nurse-led follow-up of patients after oesophageal or gastric cardia cancer surgery: a randomised trial

**DOI:** 10.1038/sj.bjc.6604811

**Published:** 2008-12-09

**Authors:** E M L Verschuur, E W Steyerberg, H W Tilanus, S Polinder, M-L Essink-Bot, K T C Tran, A van der Gaast, L P S Stassen, E J Kuipers, P D Siersema

**Affiliations:** 1 Department of Gastroenterology and Hepatology, Erasmus MC University Medical Centre Rotterdam, Rotterdam, The Netherlands; 2 Department of Public Health, Erasmus MC University Medical Centre Rotterdam, Rotterdam, The Netherlands; 3 Department of Surgery, Erasmus MC University Medical Centre Rotterdam, Rotterdam, The Netherlands; 4 Department of Medical Oncology, Erasmus MC University Medical Centre Rotterdam, Rotterdam, The Netherlands; 5 Department of Surgery, Reinier de Graaf Hospital, Delft, The Netherlands; 6 Department of Internal Medicine, Erasmus MC University Medical Centre Rotterdam, Rotterdam, The Netherlands

**Keywords:** oesophageal cancer surgery, follow-up, quality of life, patient satisfaction, costs

## Abstract

Between January 2004 and February 2006, 109 patients after intentionally curative surgery for oesophageal or gastric cardia cancer were randomised to standard follow-up of surgeons at the outpatient clinic (standard follow-up; *n*=55) or by regular home visits of a specialist nurse (nurse-led follow-up; *n*=54). Longitudinal data on generic (EuroQuol-5D, European Organization for Research and Treatment of Cancer (EORTC) QLQ-C30) and disease-specific quality of life (EORTC QLQ-OES18), patient satisfaction and costs were collected at baseline and at 6 weeks and 4, 7 and 13 months afterwards. We found largely similar quality-of-life scores in the two follow-up groups over time. At 4 and 7 months, slightly more improvement on the EQ-VAS was noted in the nurse-led compared with the standard follow-up group (*P*=0.13 and 0.12, respectively). Small differences were also found in patient satisfaction between the two groups (*P*=0.14), with spouses being more satisfied with nurse-led follow-up (*P*=0.03). No differences were found in most medical outcomes. However, body weight of patients of the standard follow-up group deteriorated slightly (*P*=0.04), whereas body weight of patients of the nurse-led follow-up group remained stable. Medical costs were lower in the nurse-led follow-up group (€2600 *vs* €3800), however, due to the large variation between patients, this was not statistically significant (*P*=0.11). A cost effectiveness acceptability curve showed that the probability of being cost effective for costs per one point gain in general quality-of-life exceeded 90 and 75% after 4 and 13 months of follow-up, respectively. Nurse-led follow-up at home does not adversely affect quality of life or satisfaction of patients compared with standard follow-up by clinicians at the outpatient clinic. This type of care is very likely to be more cost effective than physician-led follow-up.

Approximately 400 000 patients per year are diagnosed worldwide with oesophageal cancer, which makes this malignancy the eight most common cancer (Bollschweiler *et al*, 2001). The incidence of oesophageal cancer has risen remarkably over the past two decades in the Western world, due to a marked increase in the incidence of adenocarcinoma (Devesa *et al*, 1998; Botterweck *et al*, 2000). Despite recent advances in the curative treatment of oesophageal cancer (Stein and Siewert, 2004), less than 50% of patients have operable disease at presentation.

Surgery for oesophageal cancer is often accompanied by significant morbidity (Zieren *et al*, 1996; Baba *et al*, 1997; McLarty *et al*, 1997; Blazeby *et al*, 2000; De Boer *et al*, 2000, 2004; Brooks *et al*, 2002; Fagevik Olsen *et al*, 2005; Viklund *et al*, 2006). It has been reported that approximately 30% of patients will develop recurrent cancer within the first year after oesophageal resection. For these patients, the prognosis is dismal and palliation of symptoms is usually the only treatment option (Ando *et al*, 2000). Counselling and treatment of physical problems are important issues during follow-up. In addition, patients may need reassurance and emotional support during follow-up visits (Bernhard and Hurny, 1998; van ‘t Spijker, 2001).

The role of nurses in patient care has expanded (Worster *et al*, 2005). Nurses have increasingly become involved in tasks and procedures previously performed by physicians (Wright, 1997; Laurant *et al*, 2005). One area of nurses’ involvement is the development of nurse-led services in cancer care (Loftus and Weston, 2001; Cox and Wilson, 2003). We recently performed the SIREC trial, in which 209 patients were randomised to single dose (12 Gy) brachytherapy or stent placement (Homs *et al*, 2004). In this study, patients were prospectively followed by home visits of specialised research nurses. These nurses were specifically trained to support patients with incurable oesophageal cancer. They assisted patients with filling out questionnaires on quality of life, and, as a not foreseen effect, the nurses were also found to play an important role in giving advice and support to these patients (unpublished results). Therefore, we proposed that home visits by specialised nurses could be an alternative to regular outpatient clinic visits for patients who have undergone surgical treatment for oesophageal cancer. In this trial, we compared follow-up at the outpatient clinic with follow-up by home visits of a specialist nurse with respect to health-related quality of life (HRQoL), patient satisfaction and costs.

## Materials and methods

### Study population

**Figure 1:**
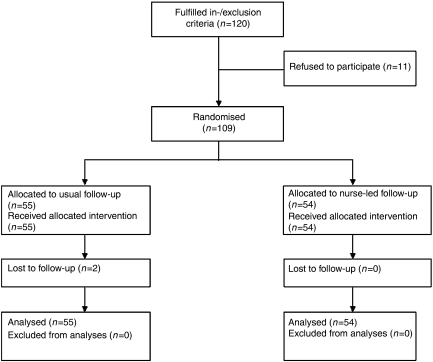
Flow chart of the study comparing standard follow-up with nurse-led follow-up in 109 patients after oesophageal cancer surgery.

We randomised patients 3 weeks after hospital discharge following intentionally curative surgery for oesophageal or gastric cardia cancer to standard follow-up at the outpatient clinic or home visits by a specialist nurse (nurse-led follow-up). Patients were excluded if they were shown to have irresectable cancer during intended oesophagectomy, if they were admitted to a nursing home after hospital discharge or if they had insufficient knowledge of the Dutch language (to fill out the questionnaires). Between January 2004 and February 2006, 120 consecutive patients were eligible to enter the trial. As 11 patients refused to participate, 109 patients were finally randomised to standard follow-up by a surgeon at the outpatient clinic (*n*=55) or home visits performed by a specialised nurse (*n*=54) (Figure 1). The major reason for unwillingness to participate was a preference for follow-up at the outpatient clinic by a physician (*n*=9). Two patients of the standard follow-up group were lost to follow-up. One patient was admitted to a nursing home during follow-up, and the other patient preferred to be evaluated in a general hospital nearby his home.

The study was approved by the Central Committee on Research Involving Human Subjects in the Netherlands. Participating centres included one university hospital (Erasmus MC – University Medical Center Rotterdam; *n*=105), and one general hospital (Reinier de Graaf Hospital Delft; *n*=4). All patients gave written informed consent for randomisation. Patients were stratified for radiation and/or chemotherapy before surgery, and for hospital. Randomisation was performed centrally by the Trial Office of the Department of Oncology, Erasmus MC Rotterdam, using computer-generated lists.

### Interventions

Nurse-led follow-up was performed by home visits of a specialist nurse with more than 10 years experience in oncological care. Didactic training included a syllabus on diagnosis and treatment of oesophageal and gastric cardia cancer, potential problems after oesophageal resection and medical-legal issues. Aspects to consider during follow-up were derived from a previous exploratory study (Verschuur *et al*, 2006). Standard follow-up was performed by a group of two senior surgeons at the outpatient clinic of the Erasmus MC Rotterdam and one senior surgeon at the Reinier de Graaf Hospital Delft. The duration of follow-up visits was recorded in both groups.

The participating surgeons as well as the specialist nurse filled out standardised case record forms. Case record forms include a list of items for assessment of patients during the follow-up visits, such as experienced problems and symptoms, body weight and the ability to eat and/or swallow using a dysphagia score (Ogilvie *et al*, 1982), graded as: 0=ability to eat a normal diet; 1=ability to eat some solids; 2=ability to eat some semisolids only; 3=ability to swallow liquids only; 4=complete dysphagia.

During follow-up, all patients were discussed during 4-weekly multidisciplinary meetings in the participating hospitals. Scheduled follow-up visits for both follow-up groups were 6 weeks, and 3, 6, 9 and 12 months after randomisation.

### Study end points

The primary outcome of the study was HRQoL; secondary outcomes included patient satisfaction and costs.

Health-related quality of life was assessed using the EuroQol-5D (Dolan, 1997) including a self-classifier with five items and a visual analogue scale (EQ-VAS) for the measurement of overall self-rated health, the oesophageal cancer-specific European Organization for Research and Treatment of Cancer (EORTC) QLQ-OES18 questionnaire (Blazeby *et al*, 2003) and the generic EORTC QLQ-C30 questionnaire (Aaronson *et al*, 1993). The EQ-5D assesses five dimensions including mobility, self-care, usual activities, pain/discomfort and anxiety/depression. For each dimension, patients mark one of three levels of severity (level 1=no problems, level 2=some/moderate problems, level 3=severe/extreme problems), which subsequently can be classified into one of 243 (3^5^) possible health status profiles. Each profile can be linked to an index score based on empirical preferences for health status from an English population (Dolan, 1997). The EQ-VAS is a 20 cm vertical visual analogue scale on which patients are asked to rate their overall health between 0 (‘worst imaginable health state’) and 100 (‘best imaginable health state’). The EORTC QLQ-OES18 incorporates five multi-item scales (dysphagia, eating, deglutition, indigestion, pain) and four single symptoms (having a dry mouth, troublesome taste, coughing and talking). The EORTC QLQ-C30 incorporates nine multi-item scales: five functional scales (physical, role, emotional, cognitive, social), three symptom scales (fatigue, nausea/vomiting, pain) and a global health/quality-of-life scale.

We developed a satisfaction questionnaire for patients as well as their spouses, as no specific validated questionnaire was available. We focused on expectations of patients, information and advice, emotional support and overall satisfaction. Replies related to satisfaction were rated as very satisfied, satisfied, dissatisfied and very dissatisfied. Patients were asked to rate their overall satisfaction on a scale from 0 to 10. A panel of three experts (a methodologist, a gastroenterologist and a gastrointestinal surgeon) had established the face validity of the questionnaire. Before its use in this trial, the questionnaire was validated in five patients.

Medical costs included costs of follow-up visits, intramural care, diagnostic procedures, additional treatments (for example, palliative treatment) and extramural care. We estimated full cost prices on the basis of real resource use from a societal perspective. Volumes of care were recorded for all patients and unit prices were determined with the micro costing method (Gold *et al*, 1996). All costs are reported in euro for the year 2006 in the Netherlands.

### Data collection

Patients were asked to complete HRQoL questionnaires before randomisation, and at 4, 7 and 13 months after randomisation. The questionnaire assessing patient satisfaction was filled out 7 months after randomisation. After reminders, patients returned 135 of the 141 (96%) questionnaires in the standard follow-up group and 144 of 147 (98%) questionnaires in the nurse-led follow-up group. The use of medical services and palliative treatment (if indicated) was assessed during follow-up visits 6 weeks, and 3, 6, 9 and 12 months after randomisation.

### Statistics

Analyses were performed on an intention-to-treat basis. We calculated that two groups of 50 patients would be sufficient for a difference of approximately 0.56 standard deviation on the standardised EuroQol-5D, with a two-sided *α*=5%, and a power of 80%.

Quality-of-life scores were evaluated with analysis of repeated measurements (Fairclough, 2002). For each scale, a model was fitted that estimated levels for all six combinations of time and follow-up group. Time and follow-up group were included as fixed factors, whereas patients were the random factor. An ANOVA test was performed for interaction between time and follow-up group. Confidence intervals around the six levels were computed based on the model. For easier interpretation of differences between randomised groups, we also estimated the average differences over time for scales on which no clear interaction was noted (*P*>0.10), using analysis of covariance with the baseline value as covariate. Clinical outcome and patient satisfaction were expressed as means±standard deviation (s.d.) and as medians, as appropriate. Body weight was evaluated with analysis of repeated measurements. Survival rates were determined using the Kaplan–Meier method. As cost data typically have a highly skewed distribution, we used non-parametric bootstrap techniques to derive a *P*-value for the differences in distribution of the direct medical costs (Thompson and Barber, 2000). Cost effectiveness was further analysed with acceptability curves. These curves showed the probability that one follow-up strategy was more cost effective compared with the other strategy for a range of values that decision makers might be willing to pay for a one point gain in the EQ-VAS for quality of life at 4 and 13 months (Fenwick *et al*, 2006).

A *P*-value <0.05 was considered statistically significant. Calculations were performed with SPSS version 11.5 (SPSS Inc., Chicago, IL, USA), and S-plus 6.0 (Insightful Inc., Seattle, WA, USA).

## Results

### Patient characteristics and functional outcome

The two patient groups were similar with respect to clinical characteristics (Table 1). The surgical procedure was a transhiatal oesophagectomy in 84 (77%) patients and a transthoracic oesophagectomy in 25 (23%). Postoperative complications at the intensive care unit included predominantly pulmonary complications (standard follow-up *n*=18 (33%) *vs* nurse-led follow-up *n*=17 (32%), *P*=0.89) and anastomotic leakage (standard follow-up *n*=3 (6%) *vs* nurse-led follow-up *n*=3 (6%), *P*=0.98). The median duration was significantly shorter in the standard follow-up group than in the nurse-led follow-up group (11 *vs* 43 min, *P*<0.01, M–W test).

All patients experienced a change in food intake pattern, resulting in the distribution of more and smaller meals over the day. During follow-up, the majority of patients were able to eat a normal diet or ate solid food with some difficulty (dysphagia score 0–1). No differences were found in dysphagia scores between the two follow-up groups during follow-up (*P*=0.20). Mean body weight of patients of the standard follow-up group deteriorated during the first year after surgery (from 73.2 kg at randomisation to 71.2 and 69.6 kg at 6 and 12 months, respectively; *P*=0.04), whereas mean body weight in the nurse-led follow-up group remained stable or slightly increased (74.5, 74.2, 75.5 kg, respectively; *P*=0.19). We found no differences in physical problems such as pain (standard follow-up *vs* nurse-led follow-up: *n*=10 (18%) *vs n*=9 (17%), 6-month visits; *P*=0.72, and *n*=5 (9%) *vs n*=5 (9%), 12-month visits; *P*=0.74) or defecation problems (*n*=8 (15%) *vs n*=6 (11%), 6-month visits; *P*=0.54), and *n*=0 (0%) *vs n*=4 (7%), 12-month visits; *P*=0.13). If specific symptoms and medical problems occurred, the specialist nurse referred patients to the outpatient clinic for medical evaluation (*n*=21, 39%). Thirty-two (28%) patients (standard follow-up *n*=15 (15%) *vs* nurse-led follow-up *n*=17 (31%); *P*=0.63) developed dysphagia (score 2–4), and these patients needed one or more (mean 3±2, range 1–8) dilations of a benign anastomotic stricture. Eleven (20%) patients of the nurse-led follow-up group and 16 (29%) patients of the standard follow-up group were diagnosed with recurrent loco regional tumour and/or metastases at 1-year survival (*P*=0.50). Of these, nine (33%) had palliative chemotherapy, whereas five (19%) patients were treated with external beam radiation therapy. Fourteen (13%) patients, seven in each follow-up group (*P*=0.41), died within the first year after surgery.

### Health-related quality of life

**Figure 2:**
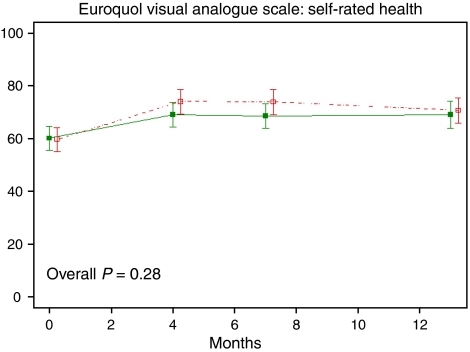
Quality of life sore after usual follow-up (*n*=55) or nurse-led follow-up (*n*=54) after oesophageal cancer surgery from the EQ-VAS. The graph shows the mean scores with 95% confidence intervals of the scale during follow-up. (- -□- - nurse-led; —▪— usual).

For all patients, the scores on the EuroQol significantly improved during follow-up, including the EQ-5D index (*P*<0.001) and the EQ-VAS for overall self-rated health (*P*<0.001). At 4 and 7 months, slightly more improvement on the EQ-VAS was noted for the nurse-led group than the standard follow-up group (mean scores 74 *vs* 69, *P*=0.13 and 0.12, respectively, Table 2 and Figure 2).

Mean EORTC QLQ-OES18 and QLQ-C30 scale scores were similar for patients of the standard follow-up group and those of the nurse-led follow-up group over time. Although not significant, better scores were found in the dysphagia scale (at 7 months, QLQ-OES18) in favour of the standard follow-up group (*P*=0.11). Similarly, in the nurse-led follow-up group, slightly better scores were found in the deglutition scale (at 13 months, QLQ-OES18; *P*=0.14), the emotional (at 4 months, QLQ-C30; *P*=0.13) and cognitive functioning scales (at 7 and 13 months, QLQ-C30; *P*=0.12 and 0.11, respectively), and global health status (at 7 months, QLQ-C30; *P*=0.12) (Table 2). For the group as a whole, a significant improvement was found in the dysphagia, eating, and indigestion scale scores (QLQ-OES18), and the fatigue, physical, role, cognitive and social functioning scales, and in global health (QLQ-C30).

### Patient satisfaction

Mean overall patient satisfaction was 8.3±1.2 for the nurse-led follow-up group compared with 7.9±1.2 for the standard follow-up group at 7 months (*P*=0.14). Spouses of patients in the nurse-led follow-up group were more satisfied with the follow-up visits then those in the standard follow-up group (mean overall rating: 8.1 *vs* 7.4; *P*=0.03). Patients and spouses in the standard follow-up group more often indicated that the visits did not fulfil their expectations (*P*=0.04 and 0.03, respectively). They frequently stated that they had expected a systematic follow-up schedule with diagnostic tests and/or procedures for the early detection of recurrent malignancy. As compared with the standard follow-up group, patients and spouses of the nurse-led follow-up group received more often advice regarding disease management (patients: *n*=45 *vs n*=37, *P*=0.04 and spouses: *n*=27 *vs n*=20; *P*=0.03). In addition, spouses of the nurse-led follow-up group more often experienced that they had an opportunity to ask questions (*P*=0.06).

### Costs

Costs of nurse-led follow-up visits were significantly lower than those of standard follow-up visits (€234 *vs* €503; *P*<0.001, Table 3). Costs for intramural care during follow-up were the highest for both types of follow-up, but differences were not statistically significant (nurse-led follow-up €1477 *vs* standard follow-up €2277; *P*=0.19). Mean hospital stay was 8.9 days for nurse-led follow-up *vs* 17.8 days for standard follow-up (*P*=0.07). Costs were similar in both follow-up groups for diagnostic procedures (nurse-led follow-up €588 *vs* standard follow-up €689; *P*=0.34), additional treatments (€182 *vs* €255; *P*=0.29) and extramural care (€111 *vs* €74; *P*=0.97). Total costs were substantially lower for nurse-led follow-up than for standard follow-up (€2592 *vs* €3789), however, due to the large variation, this difference was not statistically significant (*P*=0.11).

**Figure 3:**
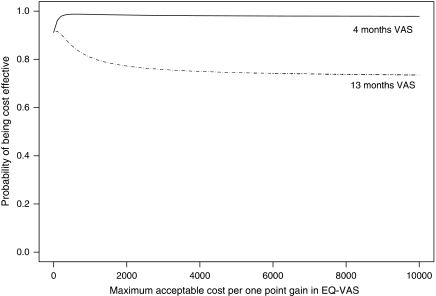
Cost effectiveness acceptability curve for nurse-led follow-up *vs* standard follow-up.

There was a 91% probability that nurse-led follow-up was cost-effective compared to standard follow-up, discarding quality-of-life effects (willingness to pay €0, Figure 3). The 4-month EQ-VAS scores were relatively high for nurse-led follow-up (mean improvement 14 *vs* 9 points, Table 2), resulting in a 98% probability that nurse-led follow-up was cost effective compared with standard follow-up at a relatively low cost of €500 per point improvement in EQ-VAS (Figure 3). The 13-month EQ-VAS scores for nurse-led follow-up had deteriorated slightly, whereas the scores for standard follow-up remained stable (mean improvement 11 *vs* 9 points). Therefore, a decision maker willing to pay €4000 or more for a one point gain on the EQ-VAS would find nurse-led follow-up cost effective with a probability of 76% (Figure 3).

## Discussion

Results from this study show that nurses can well perform follow-up of patients at home after upper gastrointestinal cancer surgery. Nurse-led follow-up had some small, statistically nonsignificant, positive effects on quality of life and satisfaction of patients and spouses. In addition, this follow-up strategy was most likely to be cost effective compared with standard follow-up.

As far as we are aware, no previous studies have reported about follow-up of cancer patients by home visits, although nurses have increasingly become involved in the care of patients with malignancies (Loftus and Weston, 2001). Results from this study are in line with findings in other studies, in which nurse-led follow-up of patients undergoing pelvic radiotherapy (Faithfull *et al*, 2001) or with lung cancer (Moore *et al*, 2002) was also reported to be effective with regard to assessment of symptoms, patient satisfaction and costs.

For some disease-specific or generic quality-of-life scores, slightly more improvement was noted for the nurse-led than the standard follow-up group. We found no differences in quality-of-life scores over time in the two follow-up groups. In agreement with other studies, the largest improvement in quality of life was seen during the first months after surgery (Zieren *et al*, 1996; De Boer *et al*, 2000, 2004; Brooks *et al*, 2002). It has previously been reported that some symptoms, such as early satiety, fatigue and diarrhoea, still persist in patients 2 years after oesophageal resection and without evidence of tumour recurrence (De Boer *et al*, 2000; Fagevik Olsen *et al*, 2005). Indeed, we found that nausea/vomiting, diarrhoea and fatigue were still present 13 months after surgery. These results confirm that a relatively extended period is required for patients to recover from oesophageal or gastric cancer surgery and to adjust to the new anatomical situation.

Assessment of patient satisfaction may provide information about the extent to which patients’ needs and expectations are addressed (Bredart *et al*, 2005). We found no differences in patient satisfaction between the nurse-led follow-up group and standard follow-up group, despite the fact that the duration of follow-up was longer in the nurse-led follow-up group than in the standard follow-up group (median: 43 *vs* 11 min). However, spouses in the nurse-led follow-up group were more satisfied with this new type of care compared with those of the standard follow-up group. Northouse *et al* (2000) found that spouses more often reported emotional distress and experienced less social support than patients. Health professionals should include family caregivers in planned programs of care. In addition, they should support both patients and spouses, not only because both have legitimate needs for support, but also because role adjustment problems in spouses may negatively affect the long-term adjustment of patients (Northouse *et al*, 2000). The results of our study support this.

If specific symptoms and medical problems occurred in the nurse-led follow-up group, patients were referred to the outpatient clinic for evaluation (*n*=21, 39%). We found no differences in occurrence of recurrent tumour growth and/or metastases, and in survival between both follow-up groups. This suggests that patients of the nurse-led follow-up group were adequately referred to a medical specialist for evaluation of symptoms and problems that occurred during follow-up, such as dysphagia or suspicion of recurrent malignancy. In the future, it may well be that curative treatment options for recurrent or metastatic disease will become available. If this is the case, a more active approach to detect recurrent or metastatic oesophageal cancer will most likely be part of the follow-up protocol of patients with resected oesophageal cancer.

Although the majority of patients were able to eat a normal diet or solid food with some difficulty, body weight of patients of the standard follow-up group deteriorated slightly (*P*=0.04), whereas body weight of patients of the nurse-led follow-up group remained stable. This can probably be explained by the fact that disease management, such as advice on food intake and diet, routinely was part of the follow-up strategy in patients of the nurse-led follow-up group and was probably less explicitly performed in the standard follow-up group.

It is remarkable that economic implications of involvement of nurses in the oncological practice have only been evaluated in a few studies (Wallace *et al*, 1999; Helgesen *et al*, 2000; Faithfull *et al*, 2001; Basnyat *et al*, 2002; Moore *et al*, 2002; Niv and Niv, 2005). We found that costs of intramural care were substantially lower in the nurse-led follow-up group, although not statistically significant. Nurse-led followed patients were less frequently admitted or visited the outpatient clinic, which translated in a reduced use of hospital-related medical services compared with the standard follow-up group. In addition, nurse-led follow-up may reconfigure care to make it more responsive to individual needs, and reduce the burden of unnecessary hospital visits and investigations for patients. Although total cost were not significantly different, acceptability curves showed that nurse-led follow-up of patients after oesophageal or gastric cardia cancer surgery was very likely a cost-effective strategy.

A limitation of this study is the limited sample size. The study was designed to test for a major difference in HRQOL (0.56 s.d., requiring >100 patients). The test was two-sided, allowing for doctors to be better or worse than the nurses. To more precisely investigate nurse-led follow-up of patients after upper gastrointestinal cancer surgery, further research is needed.

In conclusion, nurse-led follow-up at home does not adversely affect quality of life or satisfaction of patients compared with follow-up by clinicians at the outpatient clinic. Although not significant, some quality-of-life scores were in the advantage of the nurse-led follow-up group. In addition, this type of care is most likely to be more cost effective. We speculate that this type of follow-up could also be an attractive alternative to standard follow-up of patients with other types of cancer, particularly in patients in whom no curative treatment option is available for recurrent or metastastic malignancy, for example, pancreatic cancer. In addition, a nurse-led service at home may help to reduce waiting lists in hospitals and/or reduce the workload of physicians.

## Figures and Tables

**Table 1:** Clinical characteristics of 109 patients randomised to standard follow-up or nurse-led follow-up after oesophageal cancer surgery

	**Standard follow-up (*N*=55)**	**Nurse-led follow-up (*N*=54)**
Mean age; years±s.d.	61±7	61±9
*Gender; no. of patients (%)*		
Male	41 (75)	40 (74)
Female	14 (25)	14 (26)
		
*Type of reconstruction; no. of patients (%)*		
Gastric tube interposition	54 (98)	54 (100)
Colon interposition	1 (2)	0 (0)
		
*Tumour histology; no. of patients (%)*		
Adenocarcinoma	42 (76)	40 (74)
Squamous cell carcinoma	12 (22)	13 (24)
Other	1 (2)	1 (2)
		
*Prior radiation and/or chemotherapy; no. of patients (%)*		
Total	17 (31)	14 (26)
Chemotherapy	12	6
Radiation and chemotherapy	5	8
		
*Pathological staging; no. of patients (%)*		
Stage 0–I	15 (27)	13 (24)
Stage II	19 (34)	12 (22)
Stage III	8 (15)	13 (24)
Stage IV	13 (24)	16 (30)
Median dysphagia score at baseline	0	0

*P*=NS for all items.

**Table 2:** Health-related quality of life (HRQoL) during follow-up of 109 patients after oesophageal cancer surgery

	**Mean baseline score (s.d.)**			**Mean score at 4 months follow-up (s.d.)**			**Mean score at 7 months follow-up (s.d.)**			**Mean score at 13 months follow-up (s.d.)**		
**Scale**	**Standard follow-up**	**Nurse-led follow-up**	***P*-value**	**Standard follow-up**	**Nurse-led follow-up**	***P*-value**	**Standard follow-up**	**Nurse-led follow-up**	***P*-value**	**Standard follow-up**	**Nurse-led follow-up**	***P*-value**
*EuroQol (100=best)*												
EQ-5D	70 (0)	66 (0)	0.44	79 (0)	76 (0)	0.56	77 (0)	76 (0)	0.96	74 (0)	78 (0)	0.58
EuroQol VAS scale	60 (5)	60 (5)	0.89	69 (5)	74 (6)	0.13	69 (6)	74 (6)	0.12	69 (7)	71 (6)	0.66
												
*EORTC QLQ-OES18 (0=best)*												
Dysphagia scale	17 (7)	17 (7)	0.96	12 (7)	11 (7)	0.83	8 (7)	11 (7)	0.11	11 (9)	14 (9)	0.34
Eating scale	34 (10)	36 (10)	0.67	28 (10)	25 (11)	0.60	23 (11)	24 (11)	0.82	28 (13)	27 (11)	0.85
Deglutition scale	17 (8)	13 (8)	0.30	14 (9)	17 (9)	0.40	14 (9)	14 (9)	0.99	9 (11)	15 (10)	0.14
Indigestion scale	−5 (11)	−2 (11)	0.55	1 (11)	4 (11)	0.44	6 (11)	4 (12)	0.67	2 (14)	4 (12)	0.64
Pain scale	12 (5)	10 (5)	0.60	11 (5)	9 (6)	0.53	15 (6)	12 (6)	0.45	9 (7)	9 (6)	0.88
												
*Single items (0=best)*												
Having dry mouth	28 (13)	33 (14)	0.42	15 (14)	10 (15)	0.32	17 (15)	15 (15)	0.56	24 (19)	17 (16)	0.22
Troublesome taste	21 (13)	26 (12)	0.29	13 (13)	9 (13)	0.46	11 (14)	10 (14)	0.94	15 (17)	7 (14)	0.16
Troublesome coughing	33 (13)	30 (13)	0.63	23 (14)	16 (14)	0.17	23 (15)	16 (15)	0.21	21 (19)	13 (15)	0.19
Troublesome talking	22 (12)	21 (12)	0.77	13 (13)	8 (13)	0.38	15 (14)	10 (14)	0.39	13 (17)	11 (14)	0.73
												
*EORTC QLQ-C30*												
* Functional scales (100=best)*												
Physical functioning	67 (7)	64 (7)	0.44	81 (8)	80 (8)	0.83	82 (8)	81 (8)	0.79	78 (9)	82 (8)	0.45
Role functioning	46 (15)	45 (16)	0.94	69 (16)	70 (16)	0.84	71 (17)	73 (17)	0.75	69 (20)	76 (17)	0.30
Emotional functioning	79 (10)	79 (10)	0.89	76 (10)	83 (11)	0.13	77 (11)	80 (11)	0.51	79 (12)	80 (11)	0.83
Cognitive functioning	78 (9)	80 (9)	0.63	83 (10)	85 (10)	0.65	77 (10)	84 (10)	0.12	76 (12)	84 (10)	0.11
Social functioning	69 (11)	74 (11)	0.23	80 (11)	82 (11)	0.58	80 (12)	84 (12)	0.40	78 (14)	85 (12)	0.15
Global health status	61 (6)	61 (7)	0.85	73 (7)	77 (7)	0.26	72 (7)	78 (7)	0.12	71 (9)	73 (7)	0.49
												
* Symptom scales (0=best)*												
Fatigue scale	52 (12)	53 (12)	0.78	32 (13)	32 (13)	0.92	34 (13)	32 (13)	0.70	35 (15)	27 (13)	0.14
Nausea/vomiting scale	22 (9)	21 (9)	0.85	21 (10)	13 (10)	0.07	17 (10)	12 (10)	0.30	17 (12)	16 (11)	0.80
Pain scale	18 (11)	24 (11)	0.87	13 (11)	15 (12)	0.74	18 (12)	18 (12)	0.78	22 (14)	22 (12)	0.24

**Table 3:** Mean health-care use and costs (in €) per patient during follow-up after oesophageal cancer surgery

**Cost category**	**Standard follow-up (*n*=55)**	**Nurse-led follow-up (*n*=54)**	***P*-value^a^**
Follow-up visits	503	234	<0.001
Total intramural care^b^	2277	1477	0.19
Total diagnostic procedures^c^	689	588	0.34
Additional treatment^d^	255	182	0.29
Extramural care^e^	74	111	0.97
Total costs per patient	3798	2592	0.11

a Derived from 2000 bootstrap samples drawn with replacement.

b Costs include hospital stay and extra visits to outpatient clinic.

c Costs include diagnostic procedures, for example, endoscopy, X-ray, CT-scan.

d Costs include additional treatment, for example, chemotherapy, radiation therapy.

e Costs include, for example, visits to the general practitioner.
